# Hedgehog activation promotes osteogenic fates of growth plate resting zone chondrocytes through transient clonal competency

**DOI:** 10.1172/jci.insight.165619

**Published:** 2024-01-23

**Authors:** Shion Orikasa, Yuki Matsushita, Hiroaki Manabe, Michael Fogge, Zachary Lee, Koji Mizuhashi, Naoko Sakagami, Wanida Ono, Noriaki Ono

**Affiliations:** 1 University of Texas Health Science Center at Houston School of Dentistry, Houston, Texas, USA.; 2 Department of Cell Biology, Nagasaki University Graduate School of Biomedical Sciences, Nagasaki, Japan.; 3 University of Michigan School of Dentistry, Ann Arbor, Michigan, USA.

**Keywords:** Bone Biology, Stem cells, Bone development, Cartilage

## Abstract

The resting zone of the postnatal growth plate is organized by slow-cycling chondrocytes expressing parathyroid hormone-related protein (PTHrP), which include a subgroup of skeletal stem cells that contribute to the formation of columnar chondrocytes. The PTHrP–Indian hedgehog feedback regulation is essential for sustaining growth plate activities; however, molecular mechanisms regulating cell fates of PTHrP^+^ resting chondrocytes and their eventual transformation into osteoblasts remain largely undefined. Here, in a mouse model, we specifically activated Hedgehog signaling in PTHrP^+^ resting chondrocytes and traced the fate of their descendants using a tamoxifen-inducible *Pthrp-creER* line with *patched-1*–floxed and tdTomato reporter alleles. Hedgehog-activated PTHrP^+^ chondrocytes formed large, concentric, clonally expanded cell populations within the resting zone (“*patched roses*”) and generated significantly wider columns of chondrocytes, resulting in hyperplasia of the growth plate. Interestingly, Hedgehog-activated PTHrP^+^ cell descendants migrated away from the growth plate and transformed into trabecular osteoblasts in the diaphyseal marrow space in the long term. Therefore, Hedgehog activation drives resting zone chondrocytes into transit-amplifying states as proliferating chondrocytes and eventually converts these cells into osteoblasts, unraveling a potentially novel Hedgehog-mediated mechanism that facilitates osteogenic cell fates of PTHrP^+^ skeletal stem cells.

## Abstract 2

<p>PTHrP-expressing chondrocytes in the resting zone of the growth plate transform into trabecular bone osteoblasts upon Hedgehog activation.</p>

## Graphical abstract

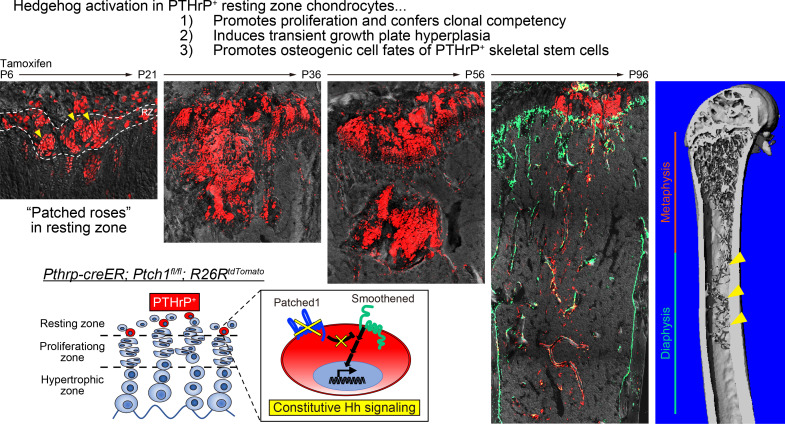

## Introduction

The growth plate — a thin disk-like cartilage located at the edges of long bones — provides the principal driving force for postnatal bone growth (1). Structurally, the growth plate is composed of 3 morphologically distinct layers of resting, proliferating, and hypertrophic zones with characteristic columns of chondrocytes with clonal origins (2, 3). The growth plate is an essential structure for endochondral bone formation, the process by which the transient cartilage is gradually replaced by the bone (1, 2). The resting zone located at the top of the postnatal growth plate houses slow-cycling chondrocytes expressing parathyroid hormone-related protein (PTHrP) (4), which provide a source of all other chondrocytes within the growth plate. These “resting” chondrocytes enter the cell cycle through asymmetric divisions, become proliferating chondrocytes, differentiate into postmitotic prehypertrophic chondrocytes that express Indian hedgehog (Ihh), become hypertrophic chondrocytes at the bottom of the growth plate, and eventually undergo either cell death because of apoptosis or transformation into trabecular bone osteoblasts in the primary spongiosa.

The resting zone of the postnatal growth plate contains populations of stem-like cells that give rise to clones of proliferating chondrocytes and determine their orientation, as first identified by autologous surgical transplantation experiments in rabbits (5) and later confirmed by in vivo genetic lineage-tracing experiments (6). The resting zone is organized by the stem cell subgroups that play important roles in long-term tissue renewal, including PTHrP^+^ (6), FoxA2^+^ (7) cells, and other slow-cycling cells that are maintained in a Wnt-inhibitory environment (8), collectively referred to as epiphyseal skeletal stem cells (9, 10). Signals from the epiphyseal stem cell niche — the secondary ossification center (SOC), composed of a variety of bone cells such as osteoblasts, osteocytes, hypertrophic chondrocytes, and hematopoietic cells — play important roles in forming these stem cells within the resting zone (11).

The PTHrP–Ihh negative feedback system is essential for maintaining activities of the growth plate. PTHrP functions as a forward signal from undifferentiated chondrocytes in the resting zone, stimulating proliferation of chondrocytes in the adjacent proliferating zone by binding to its cognate receptor PTH1R and inhibiting their terminal differentiation into hypertrophic chondrocytes (12–14). Ihh functions as a reverse signal from terminally differentiated chondrocytes in the prehypertrophic zone, stimulating proliferation of chondrocytes in the adjacent proliferating zone and their differentiation into columnar chondrocytes while increasing PTHrP expression in the resting zone (15–17). The functional interaction between PTHrP and Ihh likely plays important roles in regulating skeletal stem cell behaviors. In Hedgehog signaling, Ihh binds to its cognate receptor patched-1 (PTCH1) and relieves its inhibitory function on the G protein–coupled receptor smoothened (SMO), leading to activation of Gli transcription factors (18). Ihh released from prehypertrophic chondrocytes within the cartilage template is essential for the formation of osteoblast precursors in the perichondrium (19–23). Gli transcription factors promote osteoblast differentiation by activating BMP functions and inducing early osteoblast differentiation in a runt-related transcription factor 2–independent manner (24, 25). Therefore, Hedgehog signaling plays important roles in osteoblast differentiation.

A subset of PTHrP^+^ resting chondrocytes represents a unique type of skeletal stem cells, which are initially unipotent and later acquire multipotency at the postmitotic stage. Hypertrophic chondrocytes transform into osteoblasts and CXCL12-abundant reticular (CAR) cells as these cells exit from the growth plate (6). Chondrocyte-to-osteoblast transformation has been demonstrated by several key in vivo lineage-tracing studies (26–28). Importantly, our previous lineage-tracing study implies that the hypertrophic chondrocyte-to-osteoblast transformation is not an efficient process, as the descendants of PTHrP^+^ resting chondrocytes contribute to only a small number of osteoblasts and CAR cells in the adult bone marrow (6). The molecular mechanisms regulating this dynamic transformation remain largely unknown.

In this study, we hypothesized that Hedgehog signaling facilitates osteogenic cell fates of PTHrP^+^ resting chondrocytes through multiple mechanisms. We addressed this hypothesis by an in vivo functional cell lineage analysis using a *Pthrp-creER* line that we reported in our previous study (6). This line can exclusively mark PTHrP^+^ chondrocytes in the resting zone of the postnatal growth plate upon tamoxifen injection, without marking any other cell types in long bones. Capitalizing on this highly cell type–specific genetic tool, our goal was to define the long-term cell fate of PTHrP^+^ resting chondrocytes when Hedgehog signaling was specifically manipulated in these cells using *Ptch1*-floxed or *Smo*-floxed alleles, to activate or deactivate Hedgehog signaling, respectively. Our *Pthrp-creER*–based approach is set apart from other inducible *creER* lines such as *Prrx1-creER* (29) or *Col2a1-creER* (17, 30), both of which mark a wide variety of cells in the osteoblast and chondrocyte lineages.

Our findings overall demonstrate that Hedgehog-activated PTHrP^+^ resting chondrocytes transiently acquire clonal competency within the resting zone and induce hyperplasia of the growth plate, and subsequently, their descendants migrate away from the growth plate and efficiently transform into trabecular bone osteoblasts in the diaphyseal marrow space. Our study strengthens the link from PTHrP^+^ chondrocytes in the resting zone to osteoblasts in the trabecular bone based on a functional genetic approach, unraveling a potentially novel Hedgehog-mediated mechanism to regulate ultimate osteogenic cell fates of PTHrP^+^ skeletal stem cells.

## Results

### PTHrP^+^ resting chondrocytes lose quiescence and establish “patched roses” upon Hedgehog activation.

**Figure 1:**
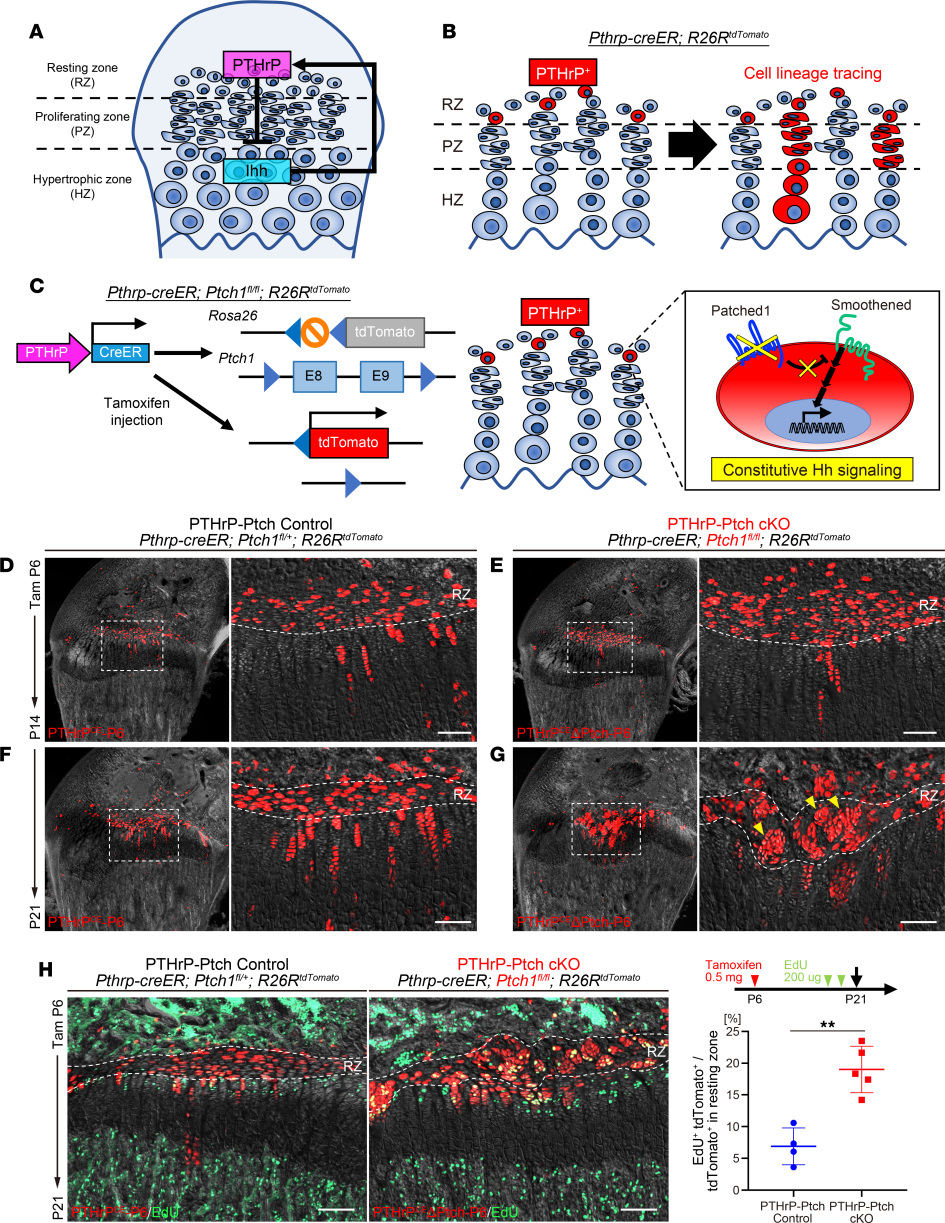
PTHrP^+^ resting chondrocytes lose quiescence and establish “patched roses” upon Hedgehog activation. (**A**) Postnatal growth plate structure with PTHrP–Ihh negative feedback system. (**B**) In vivo lineage-tracing of PTHrP^+^ resting chondrocytes using *Pthrp-creER* *R26R^tdTomato^* bigenic mice. Lineage-tracing approach. (**C**) Constitutive Hedgehog activation in PTHrP^+^ resting chondrocytes using *Pthrp-creER Ptch1 ^fl/fl^ R26R^tdTomato^* trigenic mice. (**D**–**G**) *Pthrp-creER Ptch1^fl/+^ R26R^tdTomato^* (PTHrP-Ptch Control, **D** and **F**) and *Pthrp-creER Ptch1^fl/fl^ R26R^tdTomato^* (PTHrP-Ptch cKO, **E** and **G**) distal femurs at P14 (**D** and **E**) and P21 (**F** and **G**) (pulsed at P6). Arrowheads: patched roses. Red: tdTomato. Gray: differential interference contrast (DIC). Scale bars: 100 μm. RZ, resting zone. (**H**) Cell proliferation in PTHrP-Ptch control and PTHrP-Ptch-cKO distal femur growth plate at P21 (pulsed at P6). EdU was administered by 2 serial injections (200 μg) at a 3-hour interval shortly before analysis at P21. Red: tdTomato. Green: EdU. Gray: DIC. Quantification of EdU^+^tdTomato^+^ cells. PTHrP-Ptch control (*n* = 4) and PTHrP-Ptch-cKO (*n* = 5) mice. Scale bars: 100 μm. **P < 0.01, 2-tailed, Mann-Whitney *U* test. Data are presented as mean ± SD.

We used a *Pthrp-creER* line that can specifically mark a group of PTHrP^+^ chondrocytes in the resting zone upon tamoxifen injection (6); importantly, this line does not mark any other growth plate chondrocytes in different layers, osteoblasts/cytes, or bone marrow stromal cells, enabling a highly cell type–specific lineage analysis of PTHrP^+^ resting chondrocytes in the context of physiological endochondral bone growth occurring during postnatal stages (Figure 1, A and B). Using *Pthrp-creER*, we conditionally deleted the Hedgehog cognate receptor PTCH1 using *Ptch1*-floxed alleles (31), the deletion of which causes ligand-independent activation of SMO signaling, an essential downstream effector of Hedgehog ligands (18, 32, 33). We performed in vivo functional cell lineage analysis of Hedgehog-activated descendants of PTHrP^+^ resting chondrocytes in a mosaic environment, by introducing a *cre*-responsive *R26R-tdTomato* reporter (Figure 1C). *Pthrp-creER Ptch1^fl/+^ R26R^tdTomato^* (PTHrP-Ptch control, PTHrP^CE^-P6 cells) and *Pthrp-creER Ptch1^fl/fl^ R26R^tdTomato^* (PTHrP-Ptch-cKO, PTHrP^CE^ΔPtch-P6 cells) mice were pulsed at P6 and analyzed at P14 and P21 to trace the fate of PTHrP^CE^-P6 and PTHrP^CE^ΔPtch-P6 cells by tdTomato epifluorescence.

PTHrP^+^ resting chondrocytes enter the cell cycle and start to form columnar chondrocytes after about 7 days of chase following a pulse at P6 (6). As expected, PTHrP^CE^-P6 cells formed short columns predominantly composed of fewer than 10 cells at P14 (Figure 1D). PTHrP^CE^ΔPtch-P6 cells behaved in a similar manner at this stage (Figure 1E), indicating that Hedgehog activation did not initially alter the behavior of PTHrP^+^ resting chondrocytes before the formation of the SOC. Upon further chase at P21, PTHrP^CE^-P6 cells formed long columns composed of more than 10 cells, which extended longitudinally and typically consisted of no more than 2 to 3 cells widthwise (Figure 1F). In contrast, PTHrP^CE^ΔPtch-P6 cells aberrantly expanded within the resting zone and formed “*patched roses*,” defined as concentric, seemingly clonally expanded populations of PTHrP^+^ resting chondrocytes (Figure 1G). Evaluation of cell proliferation by 5-ethynyl-2′-deoxyuridine (EdU) revealed that, while PTHrP^CE^-P6 cells predominantly remained quiescent in the resting zone, PTHrP^CE^ΔPtch-P6 cells became highly proliferative in the resting zone, seemingly in an equal proportion to those in the proliferating zone (Figure 1H). Reflecting this observation, the percentage of EdU^+^ was significantly increased in Hedgehog-activated PTHrP^CE^ΔPtch-P6 cells compared with that of control PTHrP^CE^-P6 cells (Figure 1H).

It has been previously demonstrated that Sonic Hedgehog (Shh) is abundantly expressed in the SOC (9). Our observation that Hedgehog-activated PTHrP^CE^ΔPtch-P6 cells started to form patched roses only after 2 weeks of chase prompted us to investigate the relationship between Shh^+^ cells in the SOC and PTHrP^+^ cells in the resting zone using *Pthrp-mCherry Shh-cre R26R^ZsGreen^* double reporter mice. As reported previously (6), PTHrP-mCherry^+^ cells markedly increased in the resting zone by P14, then gradually decreased toward P28, while Shh-cre-ZsGreen^+^ cells also increased in the SOC with a peak at P14 (Supplemental Figure 1A; supplemental material available online with this article; https://doi.org/10.1172/jci.insight.165619DS1). Therefore, the timing that PTHrP^+^ resting chondrocytes entered the cell cycle coincided with the expansion of Shh^+^ cells in the secondary ossification center, indicating that Shh^+^ cells may provide a cue to regulate PTHrP^+^ resting chondrocytes.

As a comparison, we also determined how uniform Hedgehog activation across all growth plate chondrocytes affects the behaviors of resting chondrocytes. *Col2a1-creER Ptch1^fl/+^ R26R^tdTomato^* (Col2a1^CE^-P6 cells) and *Col2a1-creER Ptch1^fl/fl^ R26R^tdTomato^* (Col2a1^CE^ΔPtch-P6 cells) were pulsed at P6 and analyzed at P21. Interestingly, Hedgehog activation using *Col2a1-creER* did not result in the formation of patched roses in the resting zone (Supplemental Figure 1B), indicating that Hedgehog activation needs to be specifically confined to the resting zone to confer clonal competency.

Therefore, Hedgehog activation induces loss of quiescence and aberrant clonal expansion of PTHrP^+^ chondrocytes within the resting zone, leading to the formation of clonally expanded cell clusters or patched roses, possibly in a manner responsive to Shh emanating from the SOC.

### Hedgehog activation in PTHrP^+^ resting chondrocytes causes growth plate hyperplasia.

**Figure 2:**
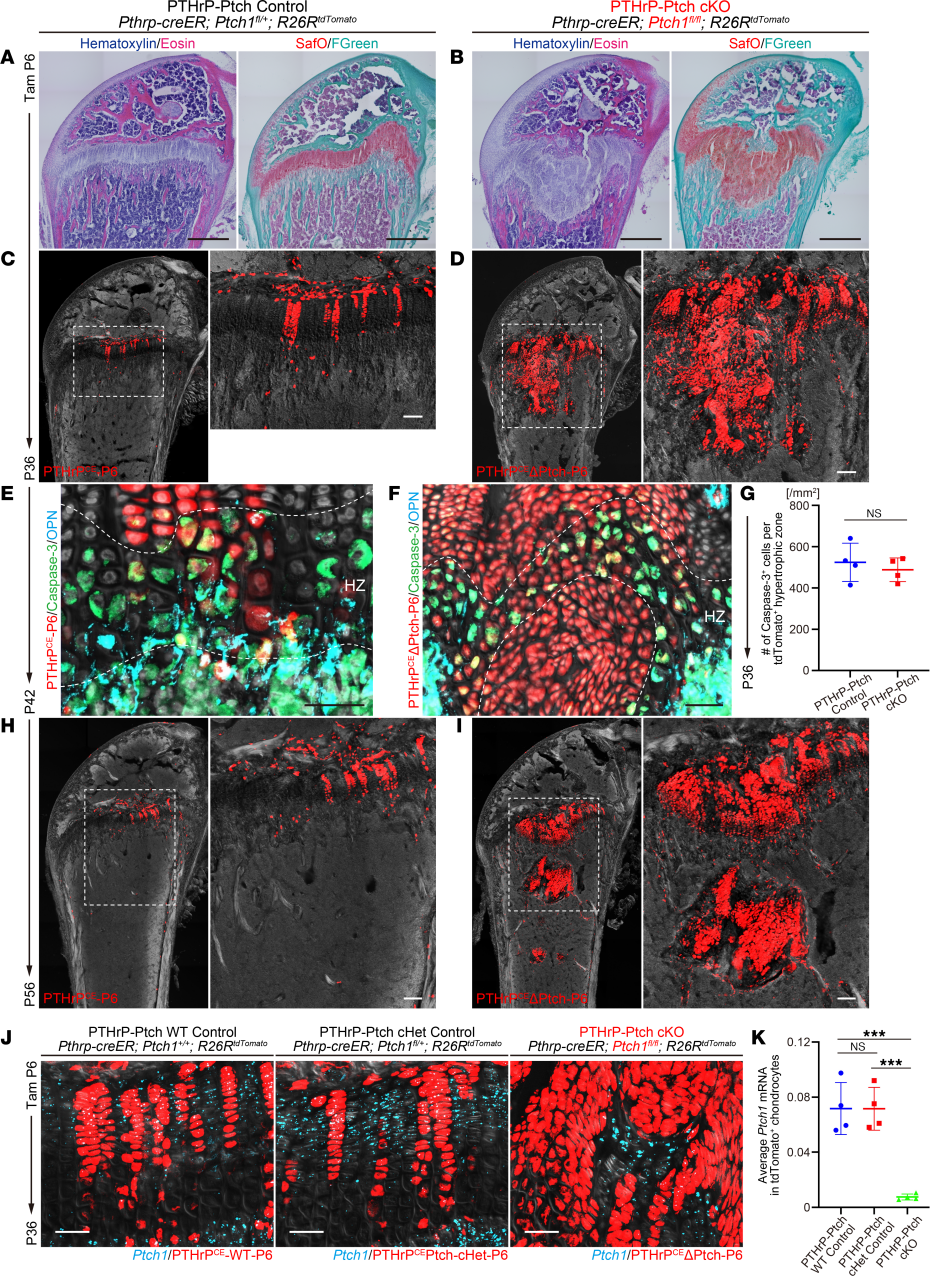
Hedgehog activation in PTHrP^+^ resting chondrocytes causes growth plate hyperplasia. (**A** and **B**) H&E and Safranin O staining of *Pthrp-creER Ptch1^fl/+^ R26R^tdTomato^* (PTHrP-Ptch Control, **A**) and *Pthrp-creER Ptch1^fl/fl^ R26R^tdTomato^* (PTHrP-Ptch cKO, **B**) distal femur at P36 (pulsed at P6). Scale bars: 500 μm. (**C**, **D**, **H**, and **I**) PTHrP-Ptch control (**C** and **H**) and PTHrP-Ptch-cKO (**D** and **I**) distal femur at P36 (pulsed at P6, **C** and **D**) and P56 (pulsed at P6, **H** and **I**). Red: tdTomato. Gray: DIC. Scale bars: 100 μm. (**E** and **F**) Apoptosis in PTHrP-Ptch control (**E**) and PTHrP-Ptch-cKO (**F**) hypertrophic zone at P42 (pulsed at P6). Immunostaining for caspase-3 and osteopontin (OPN). Green: caspase-3 (apoptosis). Red: tdTomato. Light blue: OPN. Gray: DIC. Scale bars: 50 μm. HZ, hypertrophic zone. (**G**) Quantification of Caspase-3^+^tdTomato^+^ cells among tdTomato^+^ hypertrophic chondrocytes. PTHrP-Ptch control (*n* = 4) and PTHrP-Ptch-cKO (*n* = 4) mice. (**J**) RNAscope analysis of *Ptch1^exon8–9^* in *Pthrp-creER Ptch^+/+^ R26R^tdTomato^* (PTHrP-Ptch WT Control), *Pthrp-creER Ptch1^fl/+^ R26R^tdTomato^* (PTHrP-Ptch cHet Control), and *Pthrp-creER Ptch1^fl/fl^ R26R^tdTomato^* (PTHrP-Ptch cKO) at P36 (pulsed at P6). Red: tdTomato. Light blue: *Ptch1*. Gray: DIC. Scale bars: 50 μm. (**K**) Quantification of *Ptch1^exon8–9^* mRNA levels in tdTomato^+^ chondrocytes. The ratio of *Ptch1*^+^tdTomato*^+^* area (μm^2^) to tdTomato^+^ (μm^2^) area in growth plate. *n* = 4 mice per each group. ***P < 0.001. Two-tailed, Mann-Whitney *U* test (**G**). One-way ANOVA followed by Tukey’s post hoc test (**K**). Data are presented as mean ± SD.

PTHrP^+^ resting chondrocytes contribute to the entire length of columnar chondrocytes after 1 month of chase when pulsed at P6 (6). At P36, the growth plate of control mice was entirely composed of vertically aligned columns of chondrocytes spanning over the 3 layers across the growth plate (Figure 2A and Supplemental Figure 2, A and B). In contrast, in PTHrP-Ptch-cKO mice, the boundary between the resting and proliferating zones was obliterated, wherein deranged clusters of chondrocytes bulged outward from the center of the growth plate in large masses, resulting in hyperplasia and distortion of the growth plate (Figure 2B and Supplemental Figure 2, A and B). Notably, unlike PTHrP^CE^-P6 control cells (Figure 2C), Hedgehog-activated PTHrP^CE^ΔPtch-P6 cells behaved in a substantially less linear manner, displaying deviation from the longitudinal axis associated with a loss of polarity (Figure 2D). Columns of PTHrP^CE^ΔPtch-P6 cells expanded laterally, resulting in a drastic increase in tdTomato^+^ columns within the PTHrP-Ptch-cKO growth plate (Figure 2D).

We closely examined the hypertrophic zone to evaluate chondrocyte apoptosis. In PTHrP-Ptch control mice, while a majority of PTHrP^CE^-P6 control cells were positive for caspase-3, we also noticed a small fraction of PTHrP^CE^-P6 cells that were negative for cleaved caspase-3 in the hypertrophic layer, indicative of an escape from apoptosis (Figure 2E). In PTHrP-Ptch-cKO mice, some Hedgehog-activated PTHrP^CE^ΔPtch-P6 cells were positive for caspase-3 at the disorganized chondro-osseous junction (Figure 2F), while other PTHrP^CE^ΔPtch-P6 cells also appeared to be negative for caspase-3, indicating that these cells can also escape from apoptosis. Quantification of caspase-3–positive cells revealed that the percentage of apoptotic cells was unchanged between PTHrP^CE^-P6 and PTHrP^CE^ΔPtch-P6 hypertrophic chondrocytes (Figure 2G), indicating that a drastic increase of PTHrP^CE^ΔPtch-P6 chondrocytes is due to increased proliferation but not due to decreased apoptosis.

We further examined the mutant growth plate at P56. In PTHrP-Ptch control mice, PTHrP^CE^-P6 cells were primarily located within the growth plate, with a small number of these cells also observed in the bone marrow (Figure 2H). In contrast in PTHrP-Ptch-cKO mice, clusters of PTHrP^CE^ΔPtch-P6 cells detached from the growth plate proper and formed a cartilaginous island in the metaphyseal marrow space (Figure 2I). Therefore, Hedgehog-activated PTHrP^+^ resting chondrocytes gain clonal competency and dominate the growth plate, resulting in hyperplasia of the growth plate, and then those chondrocytes detach from the growth plate and migrate toward the marrow space at later stages.

*Ptch1* heterozygosity can activate Hedgehog signaling in certain cell types. First, to define whether heterozygous loss of function of *Ptch1* can alter *Ptch1* mRNA expression, we performed RNAscope assays using a riboprobe specific to exons 8–9 of *Ptch1*, corresponding to the exons flanked by 2 *loxP* sites in the *Ptch1* locus. Littermates of *Pthrp-creER Ptch1^+/+^ R26R^tdTomato^* (PTHrP-Ptch WT control, PTHrP^CE^-WT-P6 cells), *Pthrp-creER Ptch1^fl/+^ R26R^tdTomato^* (PTHrP-Ptch-cHet control, PTHrP^CE^Ptch-cHet-P6 cells), and *Pthrp-creER Ptch1^fl/fl^ R26R^tdTomato^* (PTHrP-Ptch-cKO, PTHrP^CE^ΔPtch-P6 cells) mice were pulsed at P6 and analyzed at P36. Importantly, *Ptch1^exon8–9^* mRNA was abundantly detected in PTHrP^CE^-WT-P6 and PTHrP^CE^Ptch-cHet-P6 cells but not in PTHrP^CE^ΔPtch-P6 cells (Figure 2J). Further, in the PTHrP-Ptch-cKO growth plate, *Ptch1^exon8–9^* mRNA was abundantly detected in tdTomato-negative, hence WT, cells that did not undergo *cre-loxP* recombination by *Pthrp-creER* (Figure 2J), validating the veracity of our conditional deletion approach. Quantitative assessment of *Ptch1^exon8–9^* mRNA levels in tdTomato^+^ chondrocytes revealed that *Ptch1* was essentially abrogated in PTHrP^CE^ΔPtch-P6 cells, while no difference in *Ptch1^exon8–9^* mRNA levels was detected between PTHrP^CE^-WT-P6 and PTHrP^CE^Ptch-cHet-P6 cells (Figure 2K), indicating that *Ptch1* heterozygosity does not cause a reduction in *Ptch1* mRNA levels. Consistent with this, no difference was observed in the number of tdTomato^+^ columnar chondrocytes between the WT control and the PTHrP-Ptch-cHet growth plate (Supplemental Figure 2C), indicating that *Ptch1* heterozygosity does not enhance the column-forming capability of PTHrP^+^ resting chondrocytes.

We next asked whether inactivation of Hedgehog signaling alters in vivo dynamics of PTHrP^+^ resting chondrocytes. To achieve this, we conditionally deleted *Smo*, a major downstream effector of Hedgehog signaling, using *Smo*-floxed alleles (34), and simultaneously traced the fates of these cells using an *R26R-*tdTomato reporter allele (Supplemental Figure 3A). Littermate triple-transgenic mice with 2 corresponding genotypes — *Pthrp-creER Smo^fl/+^ R26R^tdTomato^* (PTHrP-Smo control, PTHrP^CE^-P6 cells) and *Pthrp-creER Smo^fl/fl^ R26R^tdTomato^* (PTHrP-Smo-cKO, PTHrP^CE^ΔSmo-P6 cells) mice — were pulsed at P6 with tamoxifen and analyzed at P28. PTHrP^CE^ΔSmo-P6 cells established columnar chondrocytes in a seemingly normal pattern (Supplemental Figure 3, A–C). However, the number of tdTomato^+^ columns in the PTHrP-Smo cKO was significantly less than that of the control (Supplemental Figure 3D). The bone length and overall growth plate structure were unchanged in the PTHrP-Smo cKO (Supplemental Figure 3, E and F).

Quantitative assessment revealed that *Smo* mRNA was significantly reduced in PTHrP^CE^ΔSmo-P6 cells compared with PTHrP^CE^-WT-P6 or PTHrP^CE^Smo-cHet-P6 cells (Supplemental Figure 3, G and H), demonstrating the efficacy of our *Smo* conditional inactivation. Therefore, inactivation of Hedgehog signaling in PTHrP^+^ resting chondrocytes slightly impairs their ability to establish columnar chondrocytes, without altering the overall morphology of the growth plate.

### Upregulation of PTHrP in Hedgehog-activated resting chondrocytes.

**Figure 3:**
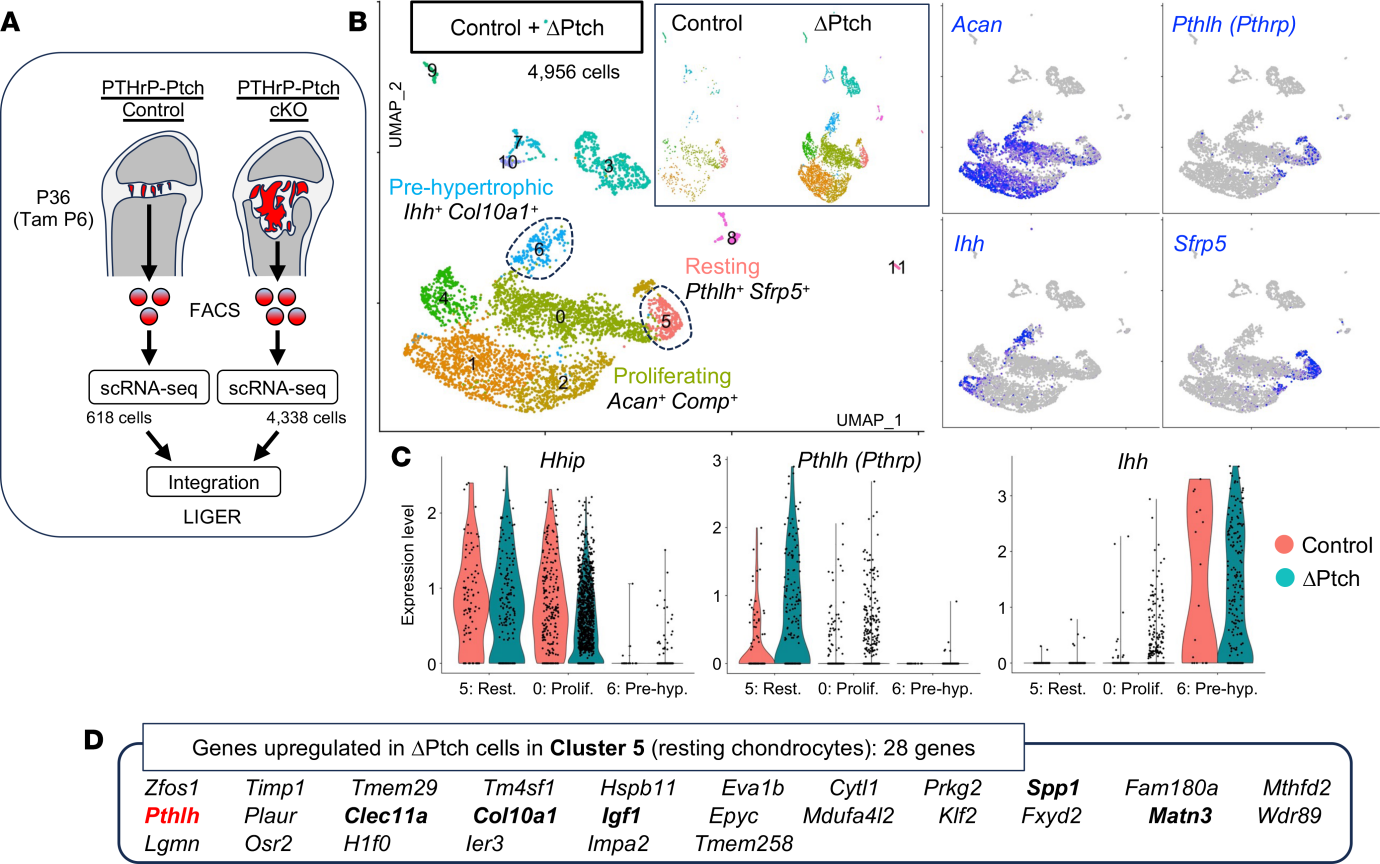
scRNA-Seq identifies the upregulation of PTHrP in Hedgehog-activated resting chondrocytes. (**A**) Diagram for LIGER data integration. scRNA-Seq data sets of sorted tdTomato^+^ single cells harvested from *Pthrp-creER Ptch1^fl/+^ R26R^tdTomato^* (PTHrP-Ptch Control, Control cell: 618 cells) and *Pthrp-creER Ptch1^fl/fl^ R26R^tdTomato^* (PTHrP-Ptch cKO, ΔPtch cell: 4,338 cells) at P36 (pulsed at P6) were merged by LIGER. (**B**) UMAP visualization of major subclusters of 2 data sets (control and ΔPtch cells) merged by LIGER. Cluster 5: resting chondrocyte (*Pthlh^+^Sfrp5^+^*), cluster 0: proliferating chondrocyte (*Acan^+^Comp^+^*), cluster 6: prehypertrophic chondrocyte (*Ihh^+^Col10a1^+^*). Right panels: feature plots of *Acan*, *Pthlh*, *Ihh*, and *Sfrp5*. Blue: high expression. (**C**) Split violin plot of *Hhip*, *Pthlh*, and *Ihh*. Red: control. Blue: ΔPtch cells in cluster 5 (resting), cluster 0 (proliferating), and cluster 6 (prehypertrophic). (**D**) List of genes upregulated in ΔPtch cells in cluster 5 (resting chondrocytes).

Subsequently, we set out to define how Hedgehog activation introduces alterations in molecular profiles in PTHrP^+^ resting chondrocytes. To achieve this goal, we performed single-cell RNA-sequencing (scRNA-Seq) analyses of PTHrP^CE^-P6 and PTHrP^CE^ΔPtch-P6 cells at P36 (pulsed at P6), which were isolated from PTHrP-Ptch-cHet control and PTHrP-Ptch-cKO growth plates, respectively, by cell sorting of tdTomato^+^ cells. The 2 scRNA-Seq data sets (control: 681 cells, and ΔPtch: 4,338 cells) were merged by linked inference of genomic experimental relationships (LIGER) (35) (Figure 3A). An initial uniform manifold approximation and projection (UMAP) analysis revealed that the cells clustered into 12 clusters, including *Pthlh(Pthrp)^+^Sfrp5^+^* resting chondrocytes (cluster 5), *Acan^+^Comp^+^* proliferating chondrocytes (cluster 0), and *Ihh^+^Col10a1^+^* prehypertrophic chondrocytes (cluster 6) (Figure 3B).

First, to define Hedgehog-responsive status of these cells, we interrogated the expression of a canonical Hedgehog-responsive gene, Hedgehog interacting protein (*Hhip*). *Pthlh^+^Sfrp5^+^* resting chondrocytes (cluster 5) expressed *Hhip* approximately to the same extent as *Acan^+^Comp^+^* proliferating chondrocytes (cluster 0) (Figure 3C). In contrast, *Ihh^+^Col10a1^+^* prehypertrophic chondrocytes did not express *Hhip* at a detectable level, indicating that both resting and proliferating chondrocytes, but not prehypertrophic chondrocytes, robustly respond to Hedgehog ligands under normal conditions.

Second, we searched for the genes that were upregulated in PTHrP^CE^ΔPtch-P6 resting chondrocytes. We identified 28 genes that were upregulated in ΔPtch cells in cluster 5, including genes with well-described functions in chondrocytes, such as *Spp1* (encoding osteopontin), *Pthlh* (encoding PTHrP), *Clec11a*, *Col10a1*, *Igf1*, and *Matn3* (Figure 3D). Therefore, the genes that we identified here, including PTHrP, might be collectively responsible for the aberrant behavior of PTHrP^CE^ΔPtch-P6 resting chondrocytes that leads to growth plate hyperplasia.

### Hedgehog-activated PTHrP^+^ descendants transform into trabecular bone osteoblasts.

**Figure 4:**
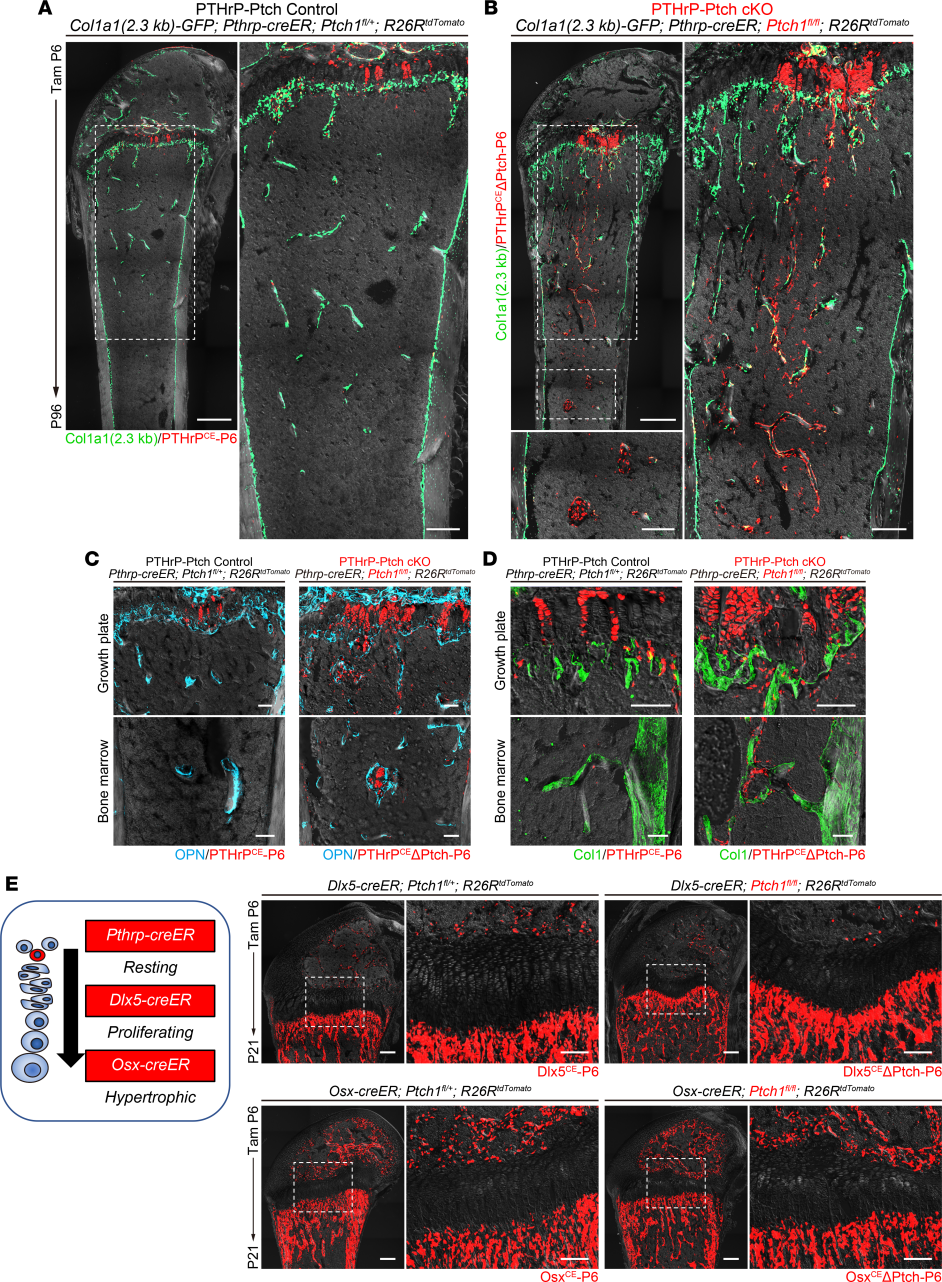
Hedgehog-activated PTHrP^+^ descendants transform into trabecular bone osteoblasts. (**A** and **B**) *Col1a1(2.3 kb)-GFP Pthrp-creER Ptch1^fl/+^ R26R^tdTomato^* (PTHrP-Ptch Control, **A**) and *Col1a1(2.3 kb)-GFP Pthrp-creER Ptch1^fl/fl^ R26R^tdTomato^* (PTHrP-Ptch cKO, **B**) distal femurs at P96 (pulsed at P6). Red: tdTomato. Green: Col1a1 (2.3 kb). Gray: DIC. Scale bars: 500 μm (low) and 200 μm (high). (**C** and **D**) Histological images of distal femurs. *Pthrp-creER Ptch1^fl/+^ R26R^tdTomato^* (PTHrP-Ptch Control) and *Pthrp-creER Ptch1^fl/fl^ R26R^tdTomato^* (PTHrP-Ptch cKO) femurs at P96 (pulsed at P6). Bone matrix proteins. Immunostaining for OPN (**C**) and Col1 (**D**). Light blue: OPN. Green: Col1. Red: tdTomato. Gray: DIC. Scale bars: 100 μm. (**E**) *Dlx5-creER Ptch1^fl/+^ R26R^tdTomato^*, *Dlx5-creER Ptch1^fl/fl^ R26R^tdTomato^*, *Osx-creER Ptch1^fl/+^ R26R^tdTomato^*, and *Osx-creER Ptch1^fl/fl^ R26R^tdTomato^* distal femurs at P21 (pulsed at P6). Red: tdTomato. Gray: DIC. Scale bars: 200 μm (low) and 100 μm (high).

Descendants of PTHrP^+^ resting chondrocytes contribute to the bone marrow and become osteoblasts and CAR stromal cells; this contribution increases for the first 3 months following a pulse at P6 (6). At P96, PTHrP^CE^-P6 control cells were predominantly present in the growth plate as columnar chondrocytes, with only a small number of cells present in the bone marrow (Figure 4A). In contrast, PTHrP^CE^ΔPtch-P6 cells contributed robustly to *Col1a1(2.3 kb)-GFP*^+^ trabecular bone osteoblasts at the same stage (Figure 4B). Strikingly, many PTHrP^CE^ΔPtch-P6 tdTomato^+^ cells were found to exist in the bone marrow and on the trabecular surface, contributing to an increase in the bone trabeculae within the PTHrP-Ptch-cKO marrow space (Figure 4B). PTHrP^CE^ΔPtch-P6 cells located on the trabecular bone surface were closely associated with bone matrix proteins OPN and type I collagen (ColI) (Figure 4, C and D). Interestingly, the PTHrP-Ptch-cKO growth plate appeared largely normal without any hyperplasia, wherein PTHrP^CE^ΔPtch-P6 cells formed columnar chondrocytes in an organized pattern (Figure 4B). Therefore, Hedgehog-activated PTHrP^+^ descendants that migrate away from the growth plate efficiently transform into trabecular bone osteoblasts in adult stages.

We further set out to determine if this increase in trabecular bone osteoblasts can be replicated by activating Hedgehog signaling in differentiated chondrocytes or osteoblast precursor cells. To this end, we utilized 2 *creER* lines, *Dlx5-creER*, which marks proliferating chondrocytes and their descendants, and *Osx-creER*, which marks hypertrophic chondrocytes and their descendants, as reported previously (6). We pulsed *Dlx5-creER Ptch1^fl/+^ R26R^tdTomato^* (Dlx5^CE^-P6 cells) and *Dlx5-creER Ptch1^fl/fl^ R26R^tdTomato^* (Dlx5^CE^ΔPtch-P6 cells), as well as *Osx-creER Ptch1^fl/+^ R26R^tdTomato^* (Osx^CE^-P6 cells) and *Osx-creER Ptch1^fl/fl^ R26R^tdTomato^* (Osx^CE^ΔPtch-P6 cells), at P6 and analyzed them at P21 (Figure 4E). Interestingly, Hedgehog activation by *Dlx5-creER* or *Osx-creER* did not ostensibly induce an increase in trabecular bones at P21. These findings highlight cell type–specific outcomes of Hedgehog activation in trabecular bone formation, supporting the notion that increased tdTomato^+^ trabecular bone osteoblasts in the PTHrP-Ptch-cKO bone marrow is a consequence of the expanded pool of tdTomato^+^ chondrocytes that serve as osteoblast precursor cells.

### Transient clonal competency of Hedgehog-activated PTHrP^+^ resting chondrocytes and their contribution to trabecular bone formation.

**Figure 5:**
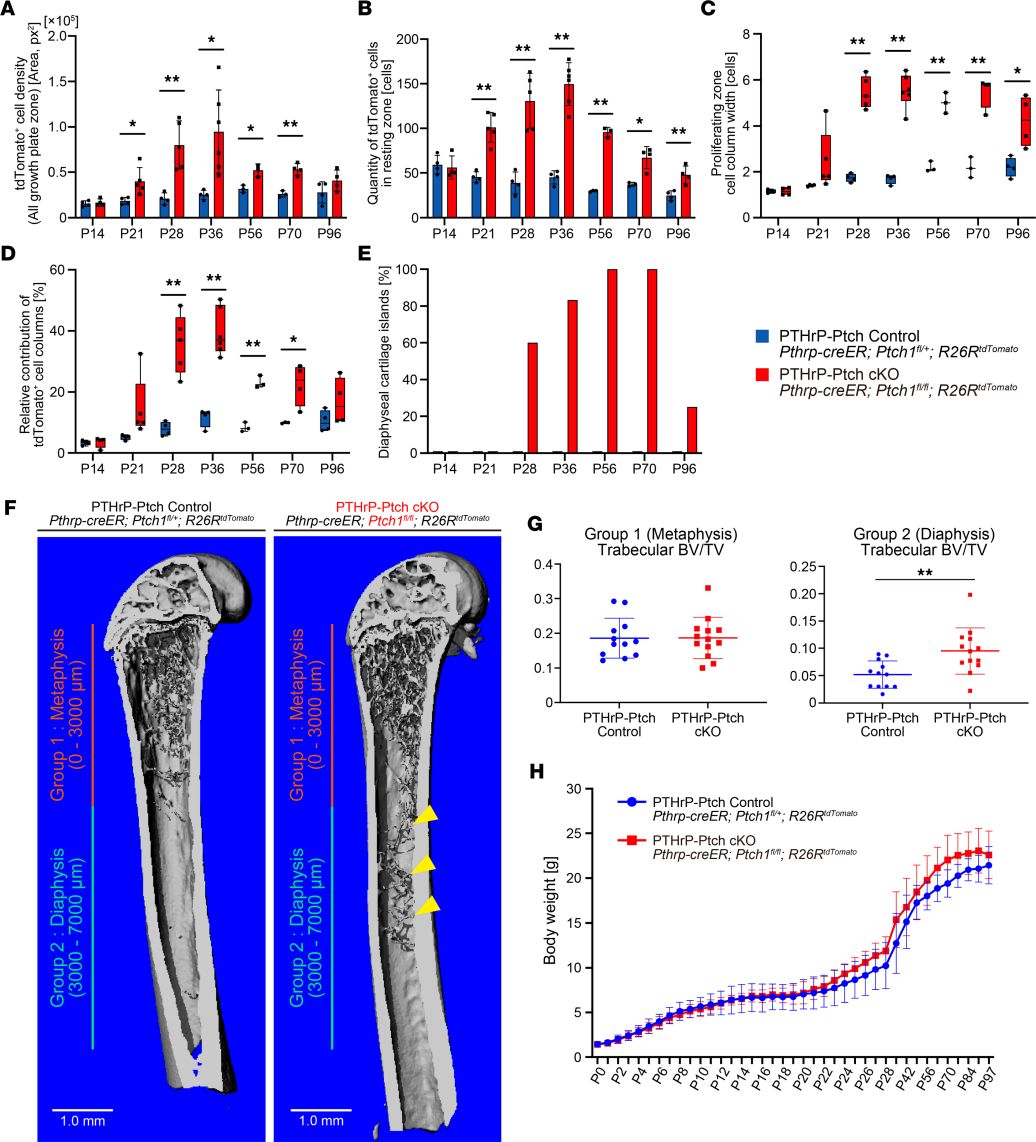
Transient clonal competency of Hedgehog-activated PTHrP^+^ resting chondrocytes and their contribution to trabecular bone formation. (**A**–**E**) Cell quantifications of *Pthrp-creER Ptch1^fl/+^ R26R^tdTomato^* (PTHrP-Ptch Control) and *Pthrp-creER Ptch1^fl/fl^ R26R^tdTomato^* (PTHrP-Ptch cKO) growth plate. (**A**) tdTomato^+^ lineage-marked cells in growth plate. tdTomato^+^ cells were quantified across all zones of the growth plate as a function of area (px^2^). (**B**) tdTomato^+^ lineage-marked cells in resting zone. Images were processed to create a selective mask excluding all cells outside of this zone prior to quantification. (**C**) Lateral cell width of columns in the proliferating zone manually counted at each time point. (**D**) Relative contribution of tdTomato^+^ columns across growth plate. A grid overlay function was used to quantify cell columns across the width of the growth plate. The number of tdTomato^+^ chondrocyte columns was calculated as a percentage of the total number of cell columns (including tdTomato^neg^ columns) across the lateral width of the growth plate. (**E**) Graph representation of the percentage of femurs containing at least 1 histological section with visible cartilage islands in diaphysis. Blue bars: PTHrP-Ptch Control (P14, 21, 28, 36, and 96: *n* = 4, P56 and 70: *n* = 3). Red bars: PTHrP-Ptch cKO (P14, 70, and 96: *n* = 4, P21 and 28: *n* = 5, P36: *n* = 6, P56: *n* = 3). (**F**) Representative 3D μCT images of PTHrP-Ptch control and PTHrP-Ptch-cKO femurs at P96 (pulsed at P6). Arrowheads: ectopic trabecular bone. Group 1: metaphysis (0–3,000 μm). Group 2: diaphysis (3,000–7,000 μm). Scale bars: 1.0 mm. (**G**) Trabecular BV/TV in group 1 (metaphysis) and group 2 (diaphysis). PTHrP-Ptch Control (*n* = 12) and PTHrP-cKO (*n* = 13) mice, including both sexes. (**H**) Body weight from P0 to P97. Blue line: PTHrP-Ptch control (*n* = 8). red: PTHrP-Ptch cKO (*n* = 9). *P < 0.05, **P < 0.01, 2-tailed Mann-Whitney *U*-test. Data are presented as mean ± SD.

To comprehensively define the kinetics of Hedgehog-activated PTHrP^+^ resting chondrocytes in the growth plate in an unbiased manner, we enumerated the number of lineage-traced PTHrP^CE^-P6 control and PTHrP^CE^ΔPtch-P6 tdTomato^+^ cells across the time points ranging from P14 to P96 using Image J/Fiji-based (NIH) cell quantification. First, we analyzed the number of tdTomato^+^ cells within the whole growth plate. In the PTHrP-Ptch control growth plate, the number of PTHrP^CE^-P6 cells gradually increased up to P56, reaching a plateau thereafter (Figure 5A, blue bars). In contrast, in the PTHrP-Ptch-cKO growth plate, PTHrP^CE^ΔPtch-P6 cells drastically and progressively increased between P21 and P36, then decreased at P56 and P70 in PTHrP-Ptch-cKO mice (Figure 5A, red bars), reaching no statistical difference at P96. A similar pattern was observed when tdTomato^+^ cells were quantified in the resting zone: PTHrP^CE^-P6 cells gradually decreased within the resting zone up to P96 (Figure 5B, blue bars), while PTHrP^CE^ΔPtch-P6 cells rapidly increased within the resting zone between P14 and P36, then decreased toward P96 (Figure 5B, red bars). These quantitative data corroborate the histological observation that PTHrP^CE^ΔPtch-P6 cells transiently gain clonal competency within the resting zone at P36.

We further examined the cell width of the tdTomato^+^ columns within the proliferating zone. PTHrP^CE^-P6 control cells established columns ~1 cell in width at P14 and P21, then columns ~2 cells in width from P28 to P96 (Figure 5C, blue bars). In contrast, in the PTHrP-Ptch cKO, PTHrP^CE^ΔPtch-P6 cells established significantly wider columns starting from P28: the cell column width of PTHrP^CE^ΔPtch-P6 increased drastically between P21 and P28, reaching a maximum average of 5–6 cells in width at P36 (Figure 5C, red bars), though this was associated with significant variability at all time points after P14. These findings support the notion that Hedgehog-activated PTHrP^CE^ΔPtch-P6 resting chondrocytes can establish larger columns of chondrocytes, contributing to the observed clonal competency and hyperplasia.

We further enumerated the relative contribution of tdTomato^+^ cell columns among all columns within the growth plate. In the PTHrP-Ptch control growth plate, PTHrP^CE^-P6 columns contributed approximately 10% of all the columns at and after P28 (Figure 5D, blue bars). In contrast in the PTHrP-Ptch-cKO growth plate, PTHrP^CE^ΔPtch-P6 columns increased substantially between P14 and P36, reaching a maximum contribution of ~40% at P36, which decreased toward P96 (Figure 5D, red bars). These quantitative results corroborate the histological observation that PTHrP^CE^ΔPtch-P6 resting chondrocytes transiently acquire clonal dominance, which is normalized in later stages.

Our histological observation in Figure 2 demonstrates that some of the PTHrP^CE^ΔPtch-P6 chondrocytes can separate from the hyperplastic growth plate and migrate toward the marrow space, ectopically forming discrete cartilage islands. We therefore quantified the occurrence of these ectopic cartilage islands within the marrow space. Importantly, no cartilage islands were observed in PTHrP-Ptch control mice at any time point (Figure 5E, blue bars). In contrast, in the PTHrP-Ptch cKO, cartilage islands first appeared at P28 and increased in prevalence over time, with 100% of all PTHrP-Ptch-cKO samples possessing cartilage islands at P56 and P70 (Figure 5E, red bars). However, interestingly, these tdTomato^+^ cartilage islands rapidly disappeared by P96, indicating that these islands are not permanent. Of note, these cartilage islands are generally located in proximity to the growth plate and later migrate toward the diaphysis over time.

The disappearance of the ectopic cartilage islands at P96 prompted us to examine quantitatively if these cartilaginous structures can transform into mineralized bone structures. To this end, we performed a 3D μCT analysis of PTHrP-Ptch control and PTHrP-Ptch-cKO bones at P96 (Figure 5F). We measured the trabecular bone volume per tissue volume (BV/TV) of 2 specific locations: group 1 (metaphysis) in the area from 0 to 3 mm away from the growth plate and group 2 (diaphysis) in the area from 3 to 7 mm away from the growth plate. Importantly, no statistically significant difference in the trabecular BV/TV was observed between PTHrP-Ptch control and PTHrP-Ptch-cKO bones in the metaphysis (group 1) (Figure 5G). However, the trabecular BV/TV of the diaphysis (group 2) was significantly increased in the PTHrP-Ptch cKO (Figure 5G), demonstrating that chondrocyte-to-osteoblast differentiation occurred in this area and resulted in mineralized tissue formation (see also Supplemental Figure 4, A and B). Indeed, apparently, contiguous structures of bone trabeculae were observed in the PTHrP-Ptch-cKO diaphyseal marrow (Figure 5F, yellow arrowheads), which may represent traces of cartilage islands transforming into mineralized structures. No difference was observed in the cortical BV/TV between the 2 groups (Supplemental Figure 4C). No significant difference was observed in body weight between the 2 groups at any time point (Figure 5H). Furthermore, the bone length of the PTHrP-Ptch-cKO femur was equivalent to that of the PTHrP-Ptch control femur at all time points, except in earlier time points of P21 (Supplemental Figure 4D), indicating that Hedgehog activation negatively affects the bone length only transiently. Therefore, the specific increase in trabecular bone mass is unlikely to be attributable to changes in systemic factors or overall bone morphology.

## Discussion

**Figure 6:**
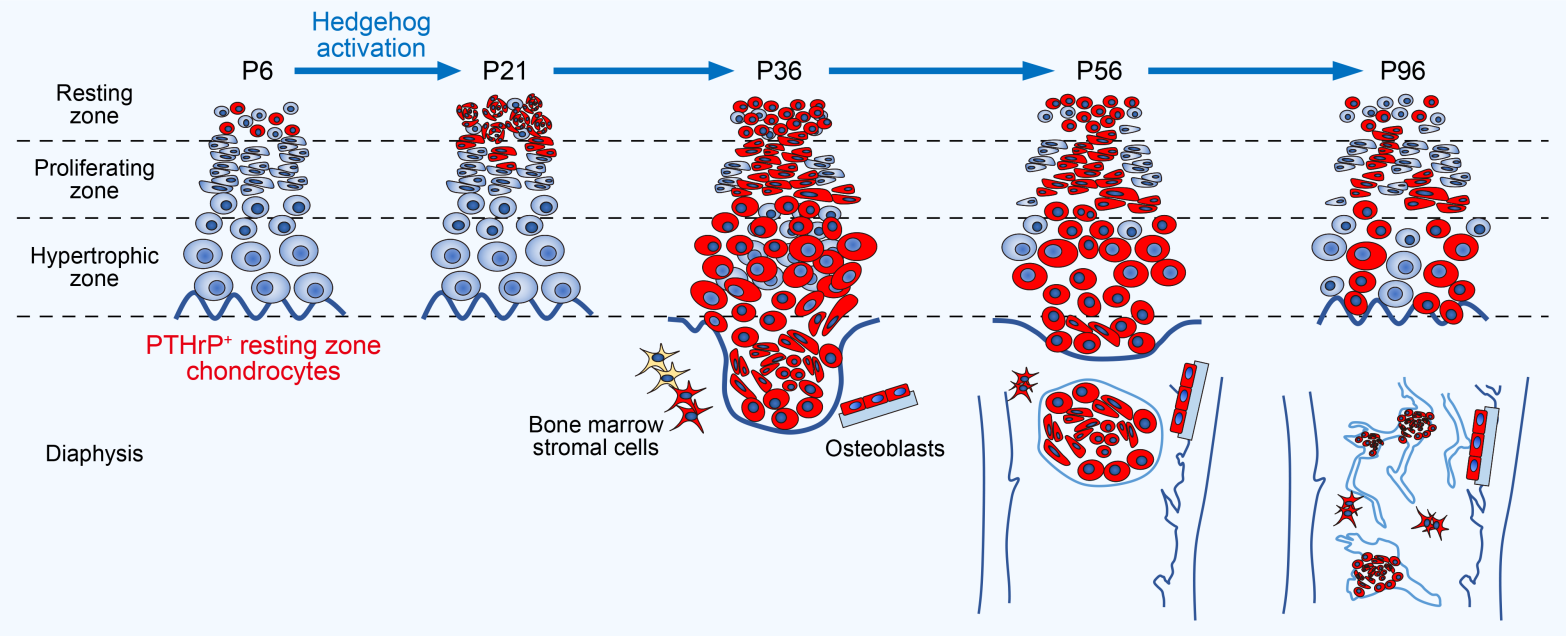
Growth plate resting zone chondrocytes acquire transient clonal competency upon hedgehog activation and transform into trabecular bone osteoblasts. Diagram of this study. In *Pthrp-creER Ptch1^fl/fl^ R26R^tdTomato^* (PTHrP-Ptch-cKO) mice, only a subset of cells in resting zone become activated for Hedgehog signaling upon tamoxifen injection. Cells marked by tdTomato are surrounded by tdTomato^–^ cells. Therefore, our resting chondrocyte–specific *Pthrp-creER* line enables a low-mosaicism analysis. PTHrP^CE^ΔPtch-P6 tdTomato^+^ cells in PTHrP-Ptch-cKO mice clonally expand and form patched roses after 2 weeks. Substantial growth plate hyperplasia and distortion are visible 1 month after pulse, and then PTHrP^CE^ΔPtch-P6 tdTomato^+^ cells separate from the growth plates and migrate to the bone marrow after 2 months. Growth plate hyperplasia in PTHrP-Ptch-cKO mice decreases to about the same level as in control mice. In addition, many PTHrP^CE^ΔPtch-P6 tdTomato^+^ cells present in bone marrow and on trabecular surfaces in PTHrP-Ptch-cKO mice are associated with an increase in trabecular bones.

Here in this study, we identified that PTHrP^+^ chondrocytes in the resting zone of the growth plate can give rise to trabecular bone–forming osteoblasts within the marrow space, when Hedgehog signaling is specifically activated in these cells (see concluding diagram, Figure 6). The resting chondrocyte–to–trabecular bone osteoblast transformation is mediated by transient amplification of proliferating chondrocytes, which might represent transit-amplifying precursors for trabecular bone–forming osteoblasts. The findings from our in vivo functional genetic cell lineage analysis provide definitive supporting evidence for 2 important processes regulating bone formation. First, hypertrophic chondrocytes can transform into osteoblasts and contribute to trabecular bone formation, and second, Hedgehog activation induces a common outcome of promoting osteoblast differentiation of early precursor cells, i.e., growth plate chondrocytes. Paracrine signals by Indian hedgehog (Ihh) released from the prehypertrophic zone may exert effects not only on cells in the adjacent perichondrium but also on chondrocytes in the adjacent proliferating zone to facilitate their osteogenic differentiation, therefore engaging multiple cellular sources to achieve endochondral bone formation (36).

We observed that Hedgehog activation transiently promotes clonal competency of PTHrP^+^ resting chondrocytes within the growth plate, resulting in the dominance of Hedgehog-activated clones during a defined period. Interestingly, Hedgehog-activated chondrocytes in the resting zone undergo an abrupt increase in clonality between 2 and 3 weeks postnatally, followed by a gradual decrease in clonal competency thereafter, indicating that a cell signaling “switch” might occur during these periods. Although it is likely that excessive proliferation of Hedgehog-activated chondrocytes is at least in part a function of cell-autonomous effects, we assume that an external physiological cue plays a role in the exaggerated proliferative response of these cells in a defined period. In the absence of such exogenous signals, we might expect Hedgehog-activated resting chondrocytes to be present in greater quantities at P14, a finding that we did not observe. Signaling switches occurring during growth plate development might exert some levels of control over the response of chondrocytes in the presence of Hedgehog activation, presumably through activation of parallel and synergistic pathways or through regulation of shared downstream inhibitors. While Hedgehog-activated chondrocytes are prone to excessive proliferation at earlier time points, these cells show neither resistance to apoptosis or inhibition of hypertrophy. The response of growth plate chondrocytes to Hedgehog activation appears to be context dependent without being restricted to a canonical action to delay chondrocyte hypertrophy or inhibit differentiation (37, 38). Our results also support a hypothesis that Hedgehog activation in PTHrP^+^ resting chondrocytes induces only a transient increase in proliferative activities, presumably due to restraint on signaling by external cues. Our findings reveal that the timing that PTHrP^+^ resting chondrocytes enter the cell cycle coincides with the expansion of Shh-expressing cells in the SOC. Although it is highly speculative, it is possible that Shh emanating from the SOC and Ihh emanating from the prehypertrophic zone coregulate PTHrP^+^ cells in the resting zone and promote their entry into the cell cycle, consistent with the concept Newton et al. proposed (9). Dissecting detailed molecular interactions between Hedgehog and other major signaling pathways in regulating skeletal stem cell behaviors within the resting zone presents an important opportunity for future studies.

Surprisingly, uniform Hedgehog activation across all growth plate chondrocytes using *Col2a1-creER* did not result in the formation of patched roses in the resting zone. Mechanisms underlying this dichotomous outcome are unknown; however, it is possible that Hedgehog-activated PTHrP^+^ resting chondrocytes need to be surrounded by non-Hedgehog-activated wild-type cells to gain clonal competency and expand abnormally within the growth plate. In contrast, Hedgehog activation across all chondrocytes in the growth plate suppresses clonal competency seemingly because of lateral inhibition.

Interestingly, Hedgehog activation in only a small number of PTHrP^+^ chondrocytes within the resting zone is sufficient to induce a cell-autonomous increase in the trabecular bone in a defined area of the diaphyseal marrow space. It is reasonable to presume chondrocyte-to-osteoblast differentiation stems from direct downstream effects of Hedgehog signaling, indirect feedback from interdependent pathways, or most likely, a combination of both. Our study demonstrates for the first time the link between PTHrP^+^ skeletal stem cells in the resting zone and trabecular bone osteoblasts in the diaphyseal marrow space. It will be interesting to examine in future studies how Hedgehog signaling spatiotemporally regulates a small number of stem cells within the resting zone and eventually exerts an impact on trabecular bone formation under physiological and pathological conditions.

In fetal bone development, classical studies have established the concept that Ihh emanating from the prehypertrophic zone stimulates chondrocyte proliferation in the resting zone (15, 16). However, none of the striking phenotypes that we observed in our study, including patched roses in the resting zone, growth plate hyperplasia, and increase in trabecular bone, has been described in these studies. Therefore, our study for the first time to our knowledge reports essential roles of Hedgehog signaling in promoting transient clonal competency of resting zone chondrocytes and their eventual chondrocyte-to-osteoblast transformation during postnatal bone growth, highlighting a novel mechanism by which Hedgehog activation induces osteoblast differentiation. Of note, this Hedgehog action is likely to be distinct from those previously identified involving perichondrial cell differentiation (19–23). We recently reported that growth plate chondrocytes and perichondrial cells represent distinct cell sources of osteoblasts during bone development and engage different mechanisms (39).

We unexpectedly found that *Smo* inactivation in PTHrP^+^ resting chondrocytes does not result in a strong phenotype in the growth plate. According to our scRNA-Seq data in Figure 3, PTHrP^+^ resting chondrocytes robustly respond to Hedgehog inputs as shown by robust *Hhip* expression. One interpretation would be that Hedgehog-responsive states of resting chondrocytes have been established long before they start forming columnar chondrocytes, therefore not requiring any additional signal through SMO. Our speculation is that excessive SMO signals because of *Ptch1* deficiency are detrimental for normal activities of PTHrP^+^ resting chondrocytes, which lead to aberrant clonal expansion and subsequent transformation into osteoblasts, though further studies are warranted to determine the relevance of our findings to normal growth plate biology.

We recognize that the growth plate hyperplasia emanating from Hedgehog activation in PTHrP^+^ resting chondrocytes has a striking resemblance to enchondroma, a benign precursor lesion that occasionally progresses to malignant chondrosarcoma (40). Our findings also support the concept that constitutive Hedgehog activation alone is not sufficient to sustain enchondromagenesis, as Hedgehog-activated cells eventually transform in trabecular bone osteoblasts. As postulated, additional signaling alterations are likely to be necessary for PTHrP^+^ resting chondrocytes to generate enchondroma.

In conclusion, our findings collectively demonstrate that Hedgehog activation drives resting zone chondrocytes into transit-amplifying states as proliferating chondrocytes and eventually converts these cells into osteoblasts, unraveling a Hedgehog-mediated mechanism that facilitates osteogenic cell fates of PTHrP^+^ skeletal stem cells. The findings from our in vivo genetic functional cell lineage analysis provide a solid foundation to understand how therapeutic intervention to the Hedgehog signaling pathway may impact a group of stem cell populations residing in the resting zone of the growth plate of growing individuals and subsequently affect bone formation.

## Methods

### Mouse strains and management.

*Pthrp-creER*, *Pthrp-mCherry* knock-in reporter (6), and *Osx-creER* (41) mice have been described previously. *Rosa26-CAG-loxP-stop-loxP-tdTomato* (Ai14: *R26R*-tdTomato, JAX007914), *Ptch1-*floxed (JAX012457), *Smo-*floxed (JAX004526), *Shh-cre* (JAX005622), *Col2a1-creER* (JAX006774), *Dlx5-creER* (JAX010705), and *Col1a1(2.3 kb)-GFP* (JAX013134) mice were acquired from The Jackson Laboratory. All procedures were conducted under the University of Texas Health Science Center at Houston’s Animal Welfare Committee (AWC), protocol AWC-21-0070, and the University of Michigan’s Institutional Animal Care and Use Committee (IACUC), protocols 5703 and 7681. All mice were housed in the animal facility accredited by the Association for Assessment and Accreditation of Laboratory Animal Care, located in the Behavioral and Biological Sciences Building of the University of Texas Health Science Center at Houston. All mice were housed in a specific pathogen–free condition and analyzed in a C57BL/6 background. Mice were housed in individually ventilated cages (Tecniplast). Access to water and food (irradiated LabDiet 5053) was ad libitum. Animal rooms were climate-controlled to provide temperatures of 21 ± 1°C, 30%–65% humidity, on a 12-hour light/12-hour dark cycle (lights on at 6:00 am Central Standard Time). For all breeding experiments, *creER* transgenes were maintained in male breeders to avoid spontaneous germline recombination. Mice were identified by micro-tattooing or ear tags. Tail biopsies of mice were lysed by a HotShot protocol (incubating the tail sample at 95°C for 30 minutes in an alkaline lysis reagent followed by neutralization) and used for PCR-based genotyping (GoTaq Green Master Mix, Promega, and Nexus X2, Eppendorf). Perinatal mice were also genotyped fluorescently (BLS miner’s lamp) whenever possible. Mice were euthanized by overdosage of carbon dioxide or decapitation under inhalation anesthesia in a drop jar (Fluriso; Isoflurane, USP; VetOne).

### Tamoxifen and induction of cre-loxP recombination for Pthrp-creER line.

Tamoxifen (MilliporeSigma T5648) was mixed with 100% ethanol until completely dissolved. Subsequently, a proper volume of sunflower seed oil (MilliporeSigma S5007) was added to the tamoxifen-ethanol mixture and rigorously mixed. The tamoxifen-ethanol-oil mixture was incubated at 60C in a chemical hood until the ethanol evaporated completely. The tamoxifen-oil mixture was stored at room temperature until use. Treatment of *Pthrp-creER Ptch1^fl/+^ R26R^tdTomato^* and *Pthrp-creER Ptch1^fl/fl^ R26R^tdTomato^* mice with various doses of tamoxifen at P6 and P9 was performed previously and determined to produce optimal responsiveness of the reporter allele at P6. Therefore, we consistently induced the lineage at P6 throughout this study.

For the EdU assay, *Pthrp-creER Ptch1^fl/+^ R26R^tdTomato^* and *Pthrp-creER Ptch1^fl/fl^ R26R^tdTomato^* mice received 500 μg of tamoxifen intraperitoneally at P6, followed by 2 serial injections of EdU (200 μg) at a 3-hour interval shortly before analysis at P21.

### Histology and immunohistochemistry.

Samples were dissected under a stereomicroscope (Nikon SMZ-800) to remove soft tissues, fixed in 4% paraformaldehyde at 4°C, and then decalcified in 15% EDTA for up to 14 days. Decalcified samples were cryoprotected in 30% sucrose/PBS solutions and then in 30% sucrose/PBS/OCT (1:1) solutions, each at least overnight at 4°C. Samples were embedded in an OCT compound (Tissue-Tek, Sakura Finetek) under a stereomicroscope and transferred on a sheet of dry ice to solidify the compound. Embedded samples were cryosectioned at 30–50 μm using a cryostat (Leica CM1850) and adhered to positively charged glass slides (Fisherbrand ColorFrost Plus, Thermo Fisher Scientific). Cryosections were stored at –20°C until use. Sections were postfixed in 4% paraformaldehyde for 15 minutes at room temperature. For immunostaining, sections were permeabilized with 0.25% Triton X-100/TBS (TBST) for 30 minutes; blocked with 3% bovine serum albumin/TBST for 30 minutes; and incubated with goat anti-OPN polyclonal antibody (1:500, R&D Systems, AF808), rabbit anti-Col1 polyclonal antibody (1:500, Cedarlane, CL50151AP), rabbit anti–caspase-3 polyclonal antibody (1:250, Promega, Thermo Fisher Scientific; PR-G7481), and Living Colors DsRed Polyclonal Antibody (1:800, Takara Bio, 632496) overnight at 4°C and subsequently with Alexa Fluor 633–conjugated donkey anti-goat IgG (1:400, Invitrogen, A21082), Alexa Fluor 647–conjugated donkey anti-rabbit IgG (1:400, Invitrogen, A31573), Alexa Fluor 546–conjugated goat anti-rabbit IgG (1:400, Invitrogen, A11035), or Alexa Fluor 488–conjugated donkey anti-goat IgG (1:400, Invitrogen, A11055) for 3 hours at room temperature. For EdU assays, sections were incubated with Alexa Fluor 488–azide (Invitrogen, A10266) for 30 minutes at 43°C using Click-iT Imaging Kit (Invitrogen, C10337). Sections were further incubated with DAPI (5 μg/mL, Invitrogen D1306) to stain nuclei prior to imaging. Stained samples were mounted in TBS with No.1.5 coverslips (Thermo Fisher Scientific).

### RNAscope in situ hybridization.

In situ hybridization was performed with RNAscope Multiplex Fluorescent Detection Reagents v2 kit (Advanced Cell Diagnostics, catalog 323110) using the following riboprobes: *Ptch1^exon8–9^* (specific to exons 8 and 9 flanked by *loxP* sites, catalog 476231) and *Smo* (catalog 318411). Briefly, sections were treated with H_2_O_2_ for 10 minutes and dehydrated with 100% ethanol for 3 minutes at room temperature. Samples were subsequently treated with RNAscope Protease III for 30 minutes at 40°C and washed with distilled water. Sections were treated with each target probe and hybridized for 2 hours, at 40°C, followed by signal hybridization by AMP and amplification by HRP-C1. TSA Vivid Fluorophore 650 (1:1,000, Advanced Cell Diagnostics, 323273) was added to label the C1 probe, and sections were treated with HRP blocker. After washing with wash buffer, sections were incubated in blocking buffer consisting of 3% bovine serum albumin/TBST for 30 minutes, at room temperature, and stained with Living Colors DsRed Polyclonal Antibody (1:800, Takara Bio, 632496) overnight at 4°C. Sections were subsequently stained with Alexa Fluor 546–conjugated goat anti-rabbit IgG (A11035, 1:400, Invitrogen) for 3 hours, at 4°C, followed by DAPI staining. Signals from RNAscope assays were quantified by using Zeiss Zen software (v3.0).

### Imaging.

Images for fixed sections were captured by an automated inverted fluorescence microscope with a structured illumination system (Zeiss Axio Observer Z1 with ApoTome.2 system) and Zen 2 (blue edition) software. Images were typically tile-scanned with a motorized stage, *Z*-stacked, and reconstructed by a maximum intensity projection function. The filter settings used were FL Filter Set 31 (excitation [Ex.] 565/30 nm, emission [Em.] 620/60 nm), Set 34 (Ex. 390/22 nm, Em. 460/50 nm), Set 38 HE (Ex. 470/40 nm, Em. 525/50 nm), Set 43 HE (Ex. 550/25 nm, Em. 605/70 nm), Set 46 HE (Ex. 500/25 nm, Em. 535/30 nm), Set 47 HE (Ex. 436/20 nm, Em. 480/20 nm), Set 50 (Ex. 640/30 nm, Em. 690/50 nm), and Set 63 HE (Ex. 572/25 nm, Em. 629/62 nm). The objectives used were Fluar 2.5×/0.12, EC Plan-Neofluar 5×/0.16, Plan-Apochromat 10×/0.45, EC Plan-Neofluar 20×/0.50, EC Plan-Neofluar 40×/0.75, and Plan-Apochromat 63×/1.40. DIC was used for objectives higher than 10×. Representative images of at least 3 independent biological samples are shown in all figures.

### Image quantification.

NIH ImageJ (Fiji, v1.52i) software was used for image manipulation and quantification. For cell quantification, serial sections (15~20 sections of 25~50 μm thickness each, total 375~1,000 μm thickness) were evaluated for each independent sample. A minimum of *n* = 4 femurs for both control and PTHrP-Ptch-cKO groups at each time point were analyzed, and values for each of approximately 20 sections per femur were averaged where appropriate.

Images of histological sections obtained using Zen 2 (blue edition) image capture software yielded raw TIFF files with 96 dpi horizontal and vertical resolution and a bit depth of 24. Images were imported into ImageJ, and a Java macro was developed to allow batch processing; image thresholding was performed to select for red (tdTomato^+^) cells, and color was subsequently set to binary. All images were cropped to an identical size of 3,960,000 pixels^2^ using a manually set rectangular region of interest (ROI) centered around the growth plate. ImageJ “Watershed” function was utilized to improve resolution by dividing large cell clusters into discrete particles.

For quantification of cell density among all growth plate zones, the ImageJ “Measure” function was utilized to determine the value of black pixels on a white background subsequent to color thresholding. Results were measured in pixels^2^.

For quantification of cell density limited to the resting zone, images were further processed to create a selective ROI excluding all cells outside of this zone. A freehand ROI was manually set for each section, and a mask was created to limit quantification to resting zone cells.

For analysis of proliferating zone cell column width, manual count of the maximum number of cells in the widest cell column (perpendicular to longitudinal axis of femur) per section was performed and results were subsequently averaged.

For analysis of relative percentage of labeled cell columns across the growth plate, 4 sections were selected per sample (sections 5, 10, 11, and 15 in numerical order) and imported to ImageJ. A grid overlay with horizontal and vertical lines spaced 20 pixels apart was applied to each image, such that 1 square cell of the grid approximated the size of 1 cell within the proliferating zone. Number of tdTomato^+^ columns within the grid was summed, and values representing the proportion of tdTomato^+^ columns out of total number of cell columns (including tdTomato^–^ columns) across the width of the growth plate were displayed as percentages.

For analysis of diaphyseal cartilage islands, all sections for both Ptch-cHet and -cKO samples at each time point were evaluated for either the presence or absence of lesions. If cartilage islands were observed either within the growth plate or marrow space of at least 1 section of a femur, that sample was deemed positive for the presence of migratory lesions.

### scRNA-Seq analysis of FACS-isolated cells.

Cell sorting was performed using a 4-laser BD FACSAria III (Ex. 407/488/561/640 nm) with a 100 μm nozzle. tdTomato^+^ cells were directly sorted into ice-cold Dulbecco’s phosphate-buffered saline (DPBS)/10% fetal bovine serum (FBS), pelleted by centrifugation at 1,500 rpm and 4°C for 10 minutes, and resuspended in 10 μL DPBS/1% FBS. Cell numbers were quantified by Countess II Automated Cell Counter (Thermo Fisher Scientific) before loading onto the Chromium Single Cell 3′ v3 microfluidics chip (10x Genomics). cDNA libraries were sequenced by NovaSeq 6000 (Illumina). The sequencing data were first preprocessed using the 10x Genomics software Cell Ranger. For alignment purposes, we generated and used a custom genome fasta and index file by including the sequences of *tdTomato-WPRE* to the mouse genome (mm10). Further downstream analysis steps were performed using the Seurat and LIGER R packages. We filtered out cells with fewer than 1,500 genes per cell and with more than 15% mitochondrial read content. The downstream analysis steps include normalization, identification of highly variable genes across the single cells, scaling based on number of unique molecular identifiers, dimensionality reduction (principal component analysis, canonical correlation analysis, and UMAP), unsupervised clustering, and discovery of differentially expressed cell type–specific markers. Differential gene expression detection to identify cell type–specific genes was performed using nonparametric Wilcoxon’s rank-sum test.

### Statistics.

Nonparametric evaluation between experimental and control samples at each time point, and for each separate parameter of data quantification, was performed using a Mann-Whitney *U* test. A *P* value of less than 0.05 was considered significant. Intra-examiner reliability (repeatability) for each method of data assessment was evaluated by repeating quantification on half of samples selected at random and comparing initial and repeated outcomes using intraclass correlation coefficient calculations.

Statistical advisement was obtained from University of Michigan Consulting for Statistics, Computing and Analytics Research. Sample size was determined on the basis of previous literature and our previous experience to give sufficient standard deviations of the mean. Investigators were not following blinded protocols during experiments and outcome assessment. Mice were used regardless of the sex. All data and images are representative of a minimum of 3 independent biological samples.

### Study approval.

All procedures were conducted under the University of Texas Health Science Center at Houston’s AWC, protocol AWC-21-0070, and the University of Michigan’s IACUC, protocols 5703 and 7681.

### Data availability.

Source data are provided with this paper. The raw data generated during and/or analyzed during the current study are available from the corresponding author on reasonable request. The scRNA-Seq data presented herein have been deposited in the National Center for Biotechnology Information’s (NCBI) Gene Expression Omnibus (GEO), and are accessible through the GEO Series accession number, SuperSeries GSE244884, including SubSeries GSE244880 and GSE244881. Reference genome mm10 (Ensembl release 93) for transcript annotation is publicly available on Ensembl, including a gtf file (http://ftp.ensembl.org/pub/release-93/gtf/mus_musculus/Mus_musculus.GRCm38.93.gtf.gz) and a fasta file (http://ftp.ensembl.org/pub/release-93/fasta/mus_musculus/dna/Mus_musculus.GRCm38.dna_sm.primary_assembly.fa.gz).

## Author contributions

SO, YM, WO, and NO designed research studies. SO, YM, HM, KM, NS, and NO conducted experiments and acquired data. SO, YM, MF, ZL, KM, NS, and NO analyzed data. WO and NO provided reagents. SO, MF, ZL, and NO wrote the manuscript.

## Supplementary Material

Supplemental data

Supporting data values

## Version 1 12/05/2023

In-Press Preview

## Version 2 01/23/2024

Electronic publication
