# Supplementary material for: Hedgehog activation promotes osteogenic fates of growth plate resting zone chondrocytes through transient clonal competency
Source: JCI Insight. 2024 Jan 23;9(2):e165619. doi: 10.1172/jci.insight.165619 (PMC10906233; doi:10.1172/jci.insight.165619)
Supplement: Supplemental data [file jciinsight-9-165619-s030.pdf]

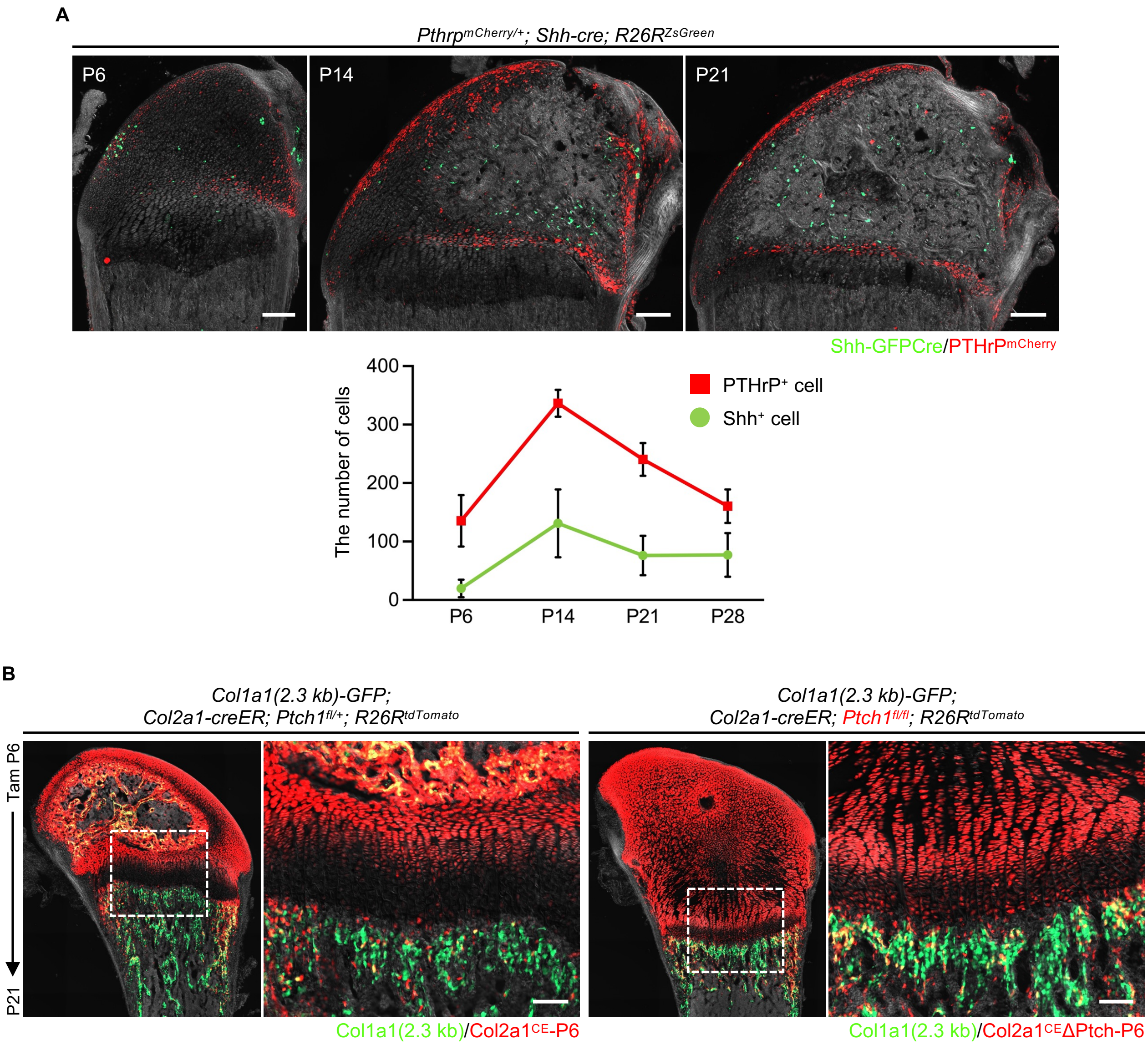

Supplemental Figure 1. Association of Shh^{+} cells in the secondary ossification center and PTHrP^{+} cells in the resting zone & uniform Hedgehog activation in growth plate chondrocytes using *Col2a1-creER*

(A) *Pthrp$^{mCherry/+}$; Shh-cre; R26R^{ZsGreen}* distal femurs and quantification at P6 (*n*=4), P14 (*n*=4), P21 (*n*=4), and P28 (*n*=3). Red: PTHrP. Green: Shh. Gray: DIC. Scale bars: 200 µm. Data are presented as mean ± s.d.

(B) *Col1a1(2.3 kb)-GFP; Col2a1-creER; Ptch$^{fl/+}$; R26R^{tdTomato}* and *Col1a1(2.3 kb)-GFP; Col2a1-creER; Ptch$^{fl/fl}$; R26R^{tdTomato}* distal femurs at P21. Red: tdTomato. Green: Col1a1(2.3 kb). Gray: DIC. Scale bars: 100 µm.

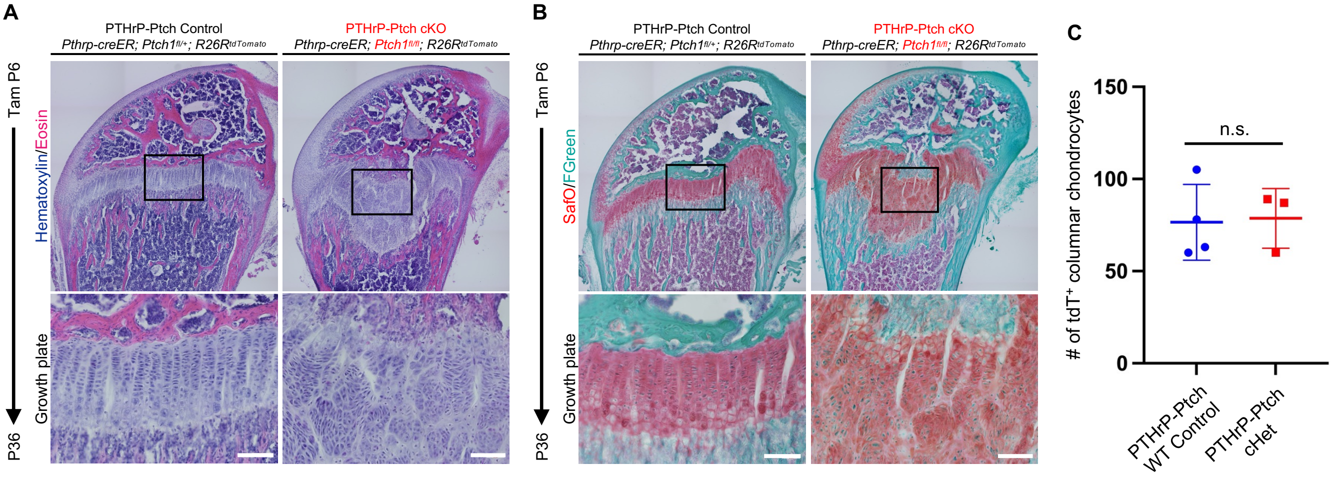

Supplemental Figure 2. Hedgehog activation in PTHrP^{+} resting chondrocytes causes growth plate hyperplasia and deviation from the longitudinal axis associated with a loss of polarity.

(A and B) H&E (**A**) and Safranin O (**B**) staining of *Pthrp-creER; Ptch1*$^{fl/+}$*; R26R*^{tdTomato} (PTHrP-Ptch Control) and *Pthrp-creER; Ptch1*$^{fl/fl}$*; R26R*^{tdTomato} (PTHrP-Ptch cKO) distal femur at P36 (pulsed at P6). Scale bars: 100 µm.
(C) Quantification of the number of tdTomato^{+} columnar chondrocytes in *Pthrp-creER; Ptch1*$^{+/+}$*; R26R*^{tdTomato} (PTHrP-Ptch WT Control, *n*=4) and *Pthrp-creER; Ptch1*$^{fl/+}$*; R26R*^{tdTomato} (PTHrP-Ptch cHet Control, *n*=3). Two-tailed, Mann-Whitney's *U*-test. Data are presented as mean ± s.d.

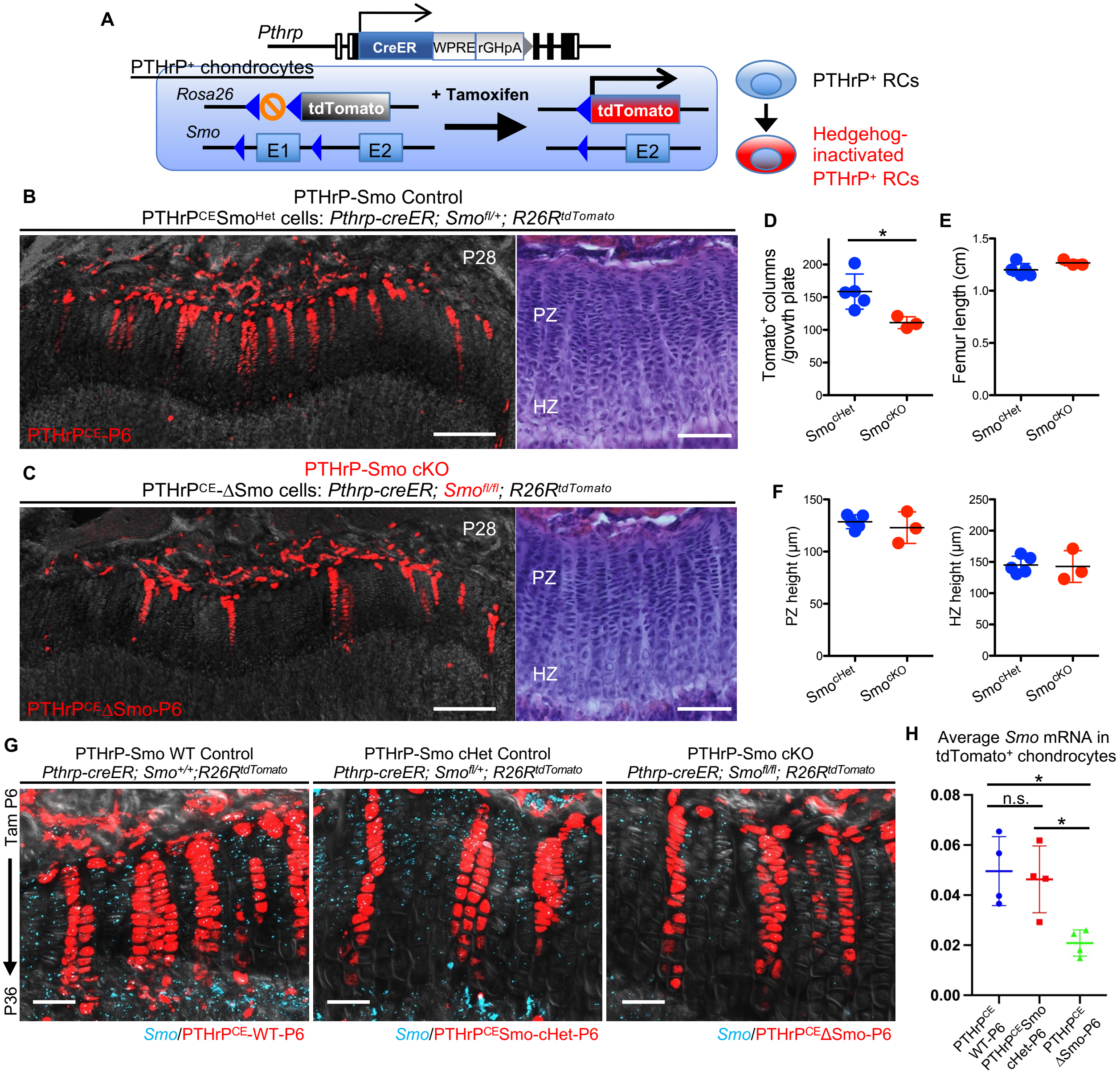

Supplemental Figure 3. Effects of Hedgehog inactivation on columnar chondrocyte formation of PTHrP⁺ resting chondrocytes.

(A) Experimental design. Hedgehog-inactivated cells can be traced by tdTomato in *Pthrp-creER; Smo*$^{fl/fl}$*; R26R*^{tdTomato} mice. RC: resting chondrocyte.

(B and C) *Pthrp-creER; Smo*$^{fl/+}$*; R26R*^{tdTomato} (PTHrP-Smo Control, **B**) and *Pthrp-creER; Smo*$^{fl/fl}$*; R26R*^{tdTomato} (PTHrP-Smo cKO, **C**) distal femur growth plates at P28, pulsed at P6. Red in (**B**): PTHrP^{CE}Smo^{Het}-tdTomato⁺ cells. Red in (**C**): PTHrP^{CE}ΔSmo-tdTomato⁺ cells. Right panels: H&E staining. PZ: proliferating zone. HZ: hypertrophic zone. Scale bars: 200 µm (left), 100 µm (right).

(D-F) Quantification. The number of tdTomato⁺ columns in growth plates (**D**), femur length (**E**), and the height of proliferating (**F**, left panel) and hypertrophic zone (**F**, right panel). *n*=5 mice for PTHrP-Smo Control, *n*=3 mice for PTHrP-Smo cKO.

(G) RNAscope analysis of *Smo* in *Pthrp-creER; Smo*$^{+/+}$*; R26R*^{tdTomato} (PTHrP-Smo WT Control), *Pthrp-creER; Smo*$^{fl/+}$*; R26R*^{tdTomato} (PTHrP-Smo cHet Control) and *Pthrp-creER; Smo*$^{fl/fl}$*; R26R*^{tdTomato} (PTHrP-Smo cKO) at P36 (pulsed at P6). Light blue: *Smo*. Red: tdTomato. Gray: DIC. Scale bars: 50 µm.

(H) Quantification of *Smo* mRNA levels in tdTomato⁺ chondrocytes. The ratio of *Smo*⁺tdTomato⁺ area [µm^2] to tdTomato⁺ [µm^2] area in growth plate. *n*=4 mice per each group. *p<0.05, two-tailed, one-way ANOVA followed by Tukey's post-hoc test. Two-tailed, Mann-Whitney's *U*-test. Data are presented as mean ± s.d.

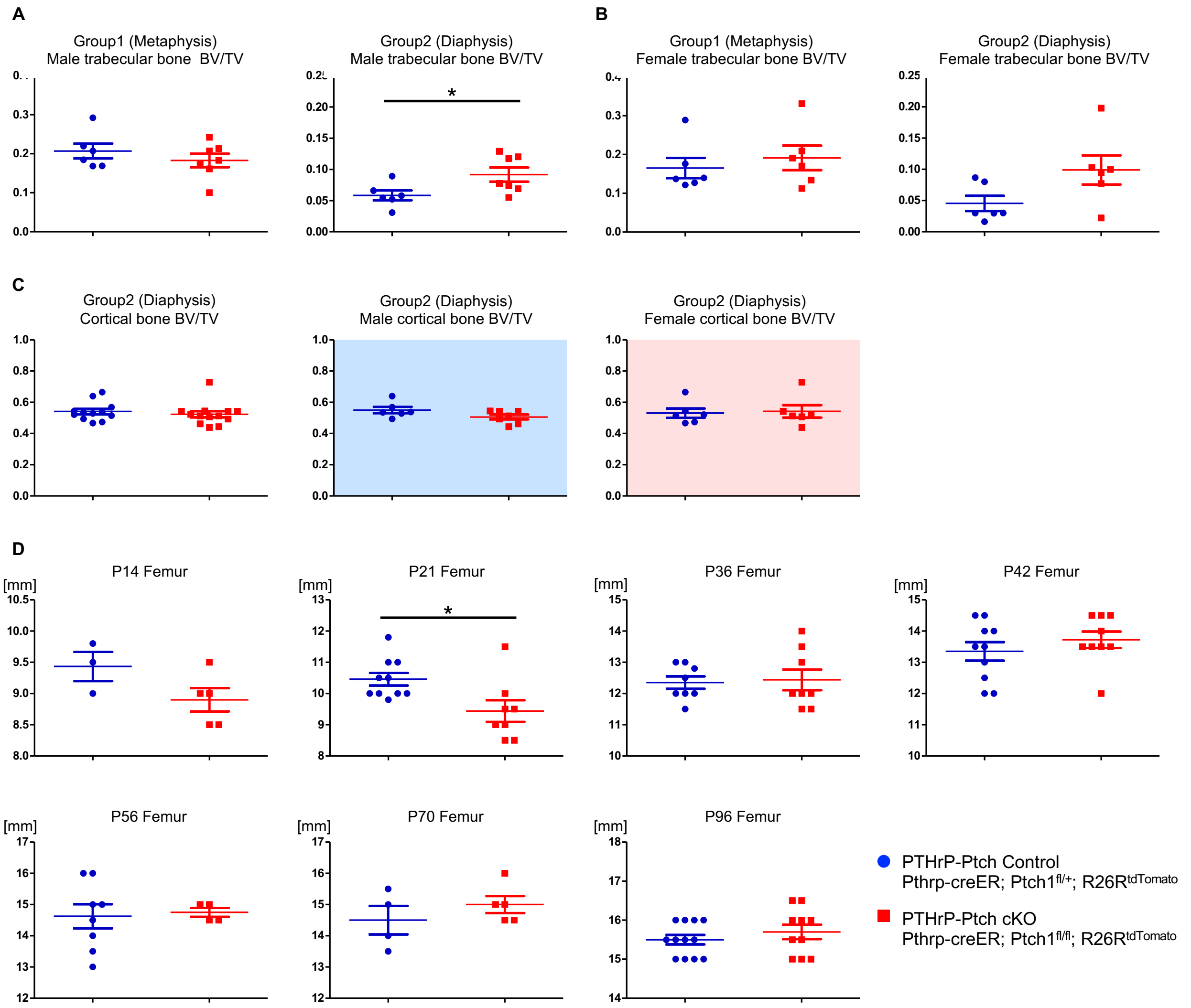

Supplemental Figure 4. Three-dimensional microCT analysis of PTHrP$^+$ Ptch1 conditional mutant bone.

(A-C) 3D-μCT analysis of *Pthrp-creER; Ptch1$^{fl/+}$; R26R^{tdTomato}* (PTHrP-Ptch Control) and *Pthrp-creER; Ptch1$^{fl/fl}$; R26R^{tdTomato}* (PTHrP-Ptch cKO) femurs at P96 (pulsed at P6). Trabecular bone BV/TV in male (PTHrP-Ptch Control: n=6, PTHrP-Ptch cKO: n=7, **A**) and female (n=6, **B**) mice, in Group 1 (metaphysis) (left) and Group 2 (diaphysis) (right). (**C**) Cortical bone BV/TV in Group 2 (diaphysis). PTHrP-Ptch Control (n=12) and PTHrP-Ptch cKO (n=13) mice, including both sexes.

(D) Femur length of PTHrP-Ptch Control (P14: n=3, P21 and 42: n=10, P36 and 56: n=8, P70: n=4, P96: n=12) and PTHrP-Ptch cKO (P14 and 70: n=5, P21 and 36: n=8, P42: n=9, P56: n=4, P96: n=10) mice pulsed at P6. Blue: PTHrP-Ptch Control. Red: PTHrP-Ptch cKO. *p<0.05, two-tailed, Mann-Whitney's U-test. Data are presented as mean ± s.d.
